# Supplementary figures and images for: Identification and comparison of circular RNAs in preeclampsia
Source: PeerJ. 2021 Apr 20;9:e11299. doi: 10.7717/peerj.11299 (PMC8063878; doi:10.7717/peerj.11299)

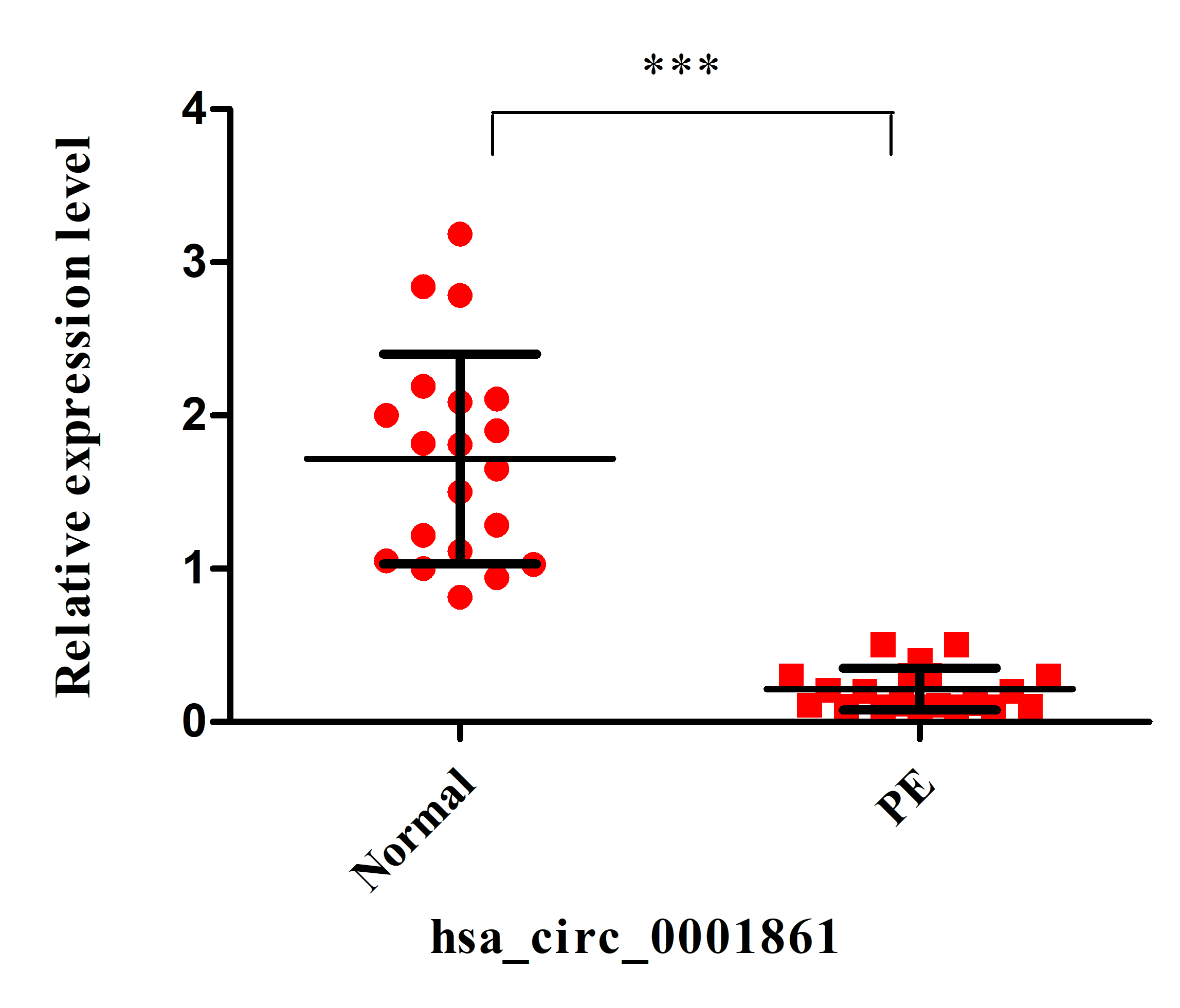

Supplement: Supplemental Information 1 — These data were analyzed for full transcriptome sequencing of patients with preeclampsia and the normal group. [file peerj-09-11299-s001.zip › down-regulated/hsa_circ_0001861.tif]

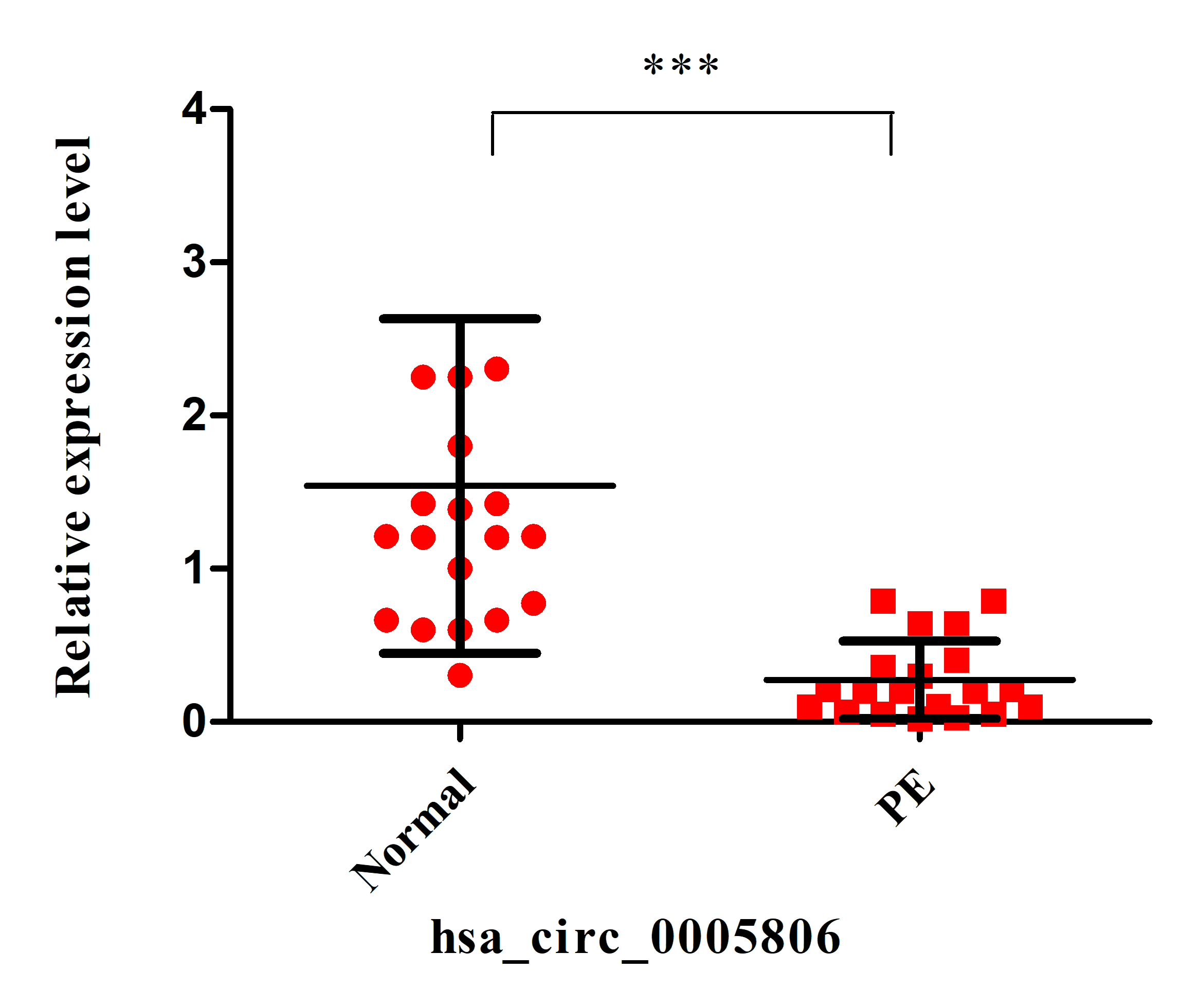

Supplement: Supplemental Information 1 — These data were analyzed for full transcriptome sequencing of patients with preeclampsia and the normal group. [file peerj-09-11299-s001.zip › down-regulated/hsa_circ_0005806.tif]

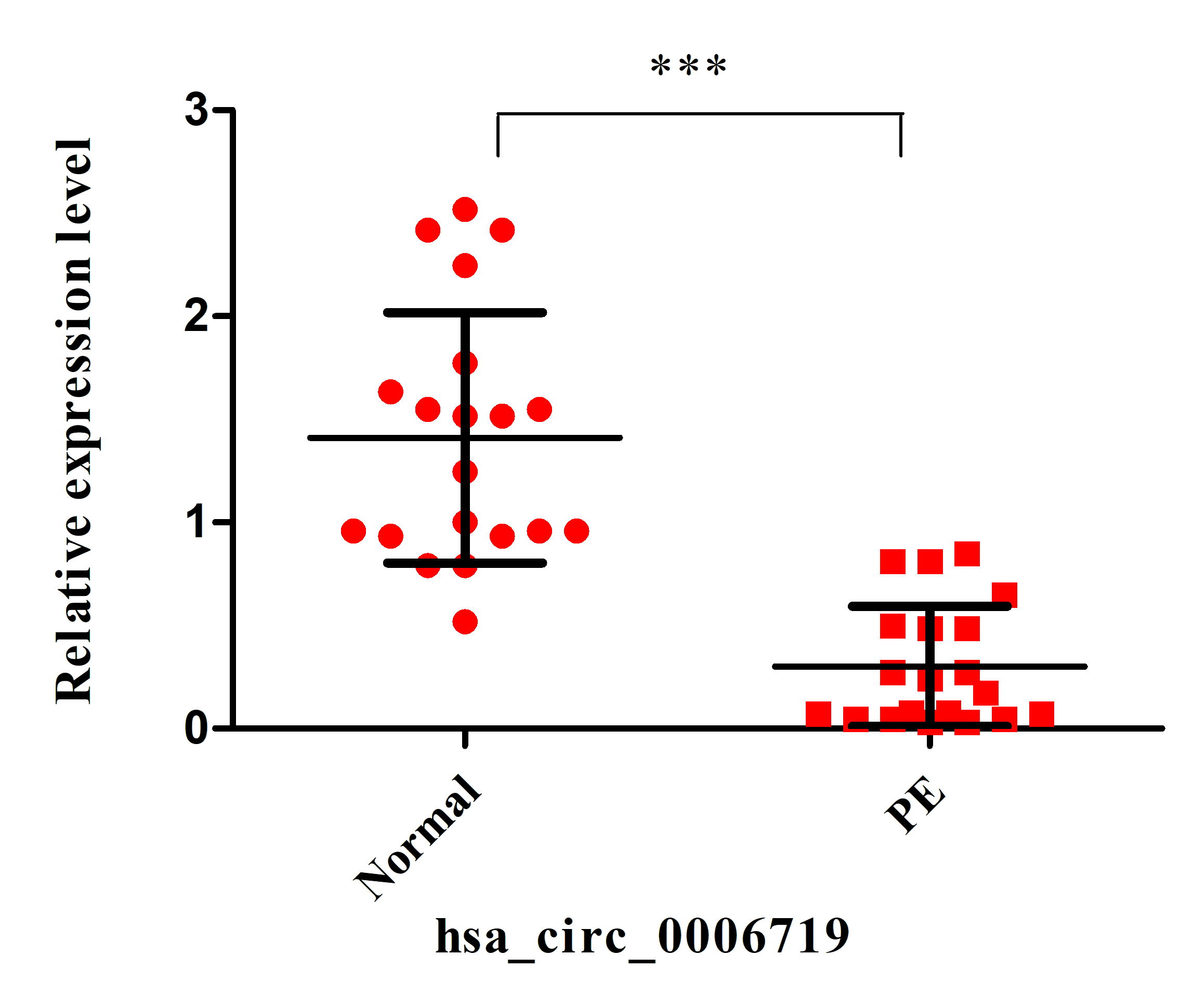

Supplement: Supplemental Information 1 — These data were analyzed for full transcriptome sequencing of patients with preeclampsia and the normal group. [file peerj-09-11299-s001.zip › down-regulated/hsa_circ_0006719.tif]

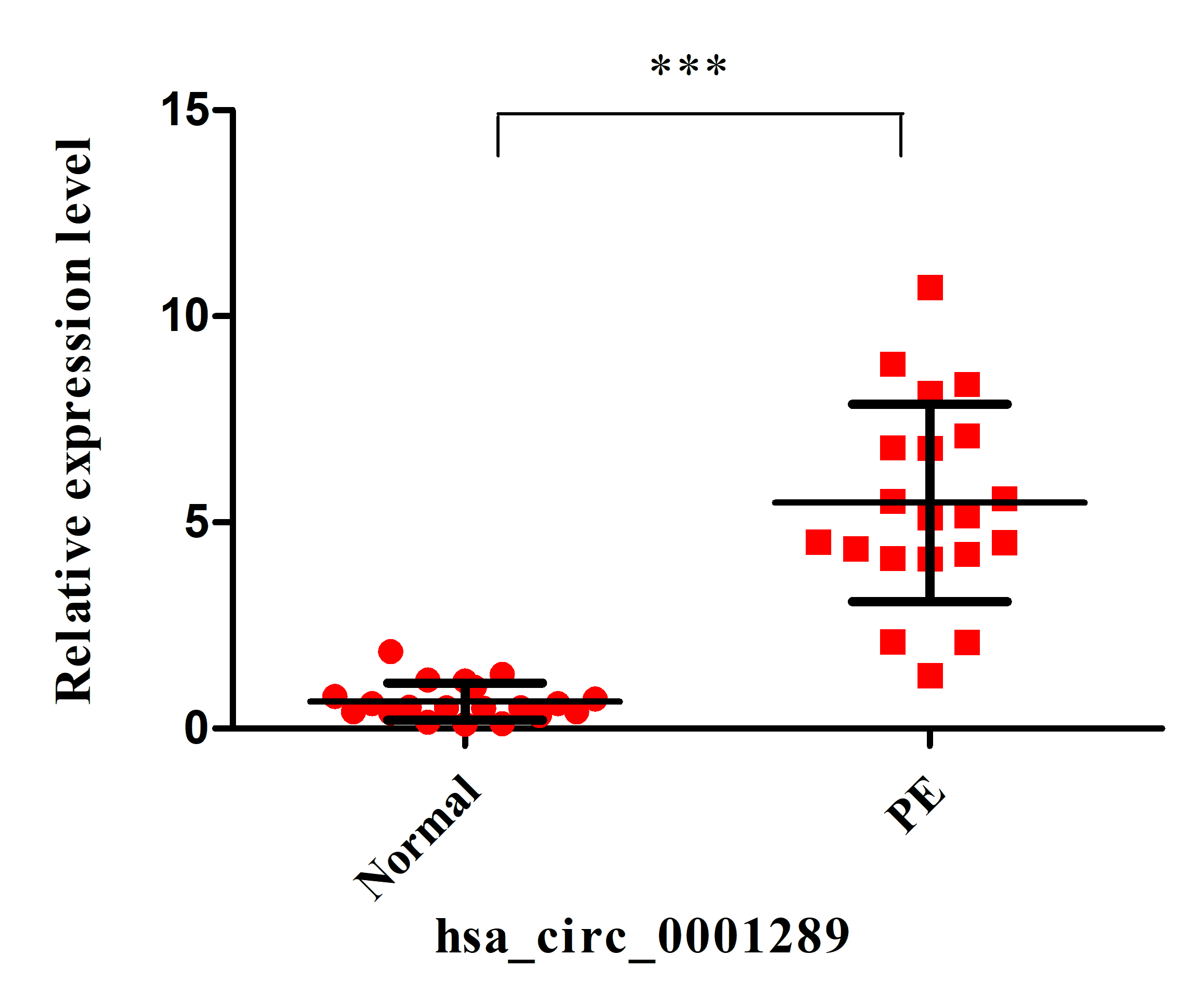

Supplement: Supplemental Information 1 — These data were analyzed for full transcriptome sequencing of patients with preeclampsia and the normal group. [file peerj-09-11299-s001.zip › up-regulated/hsa_circ_0001289.tif]

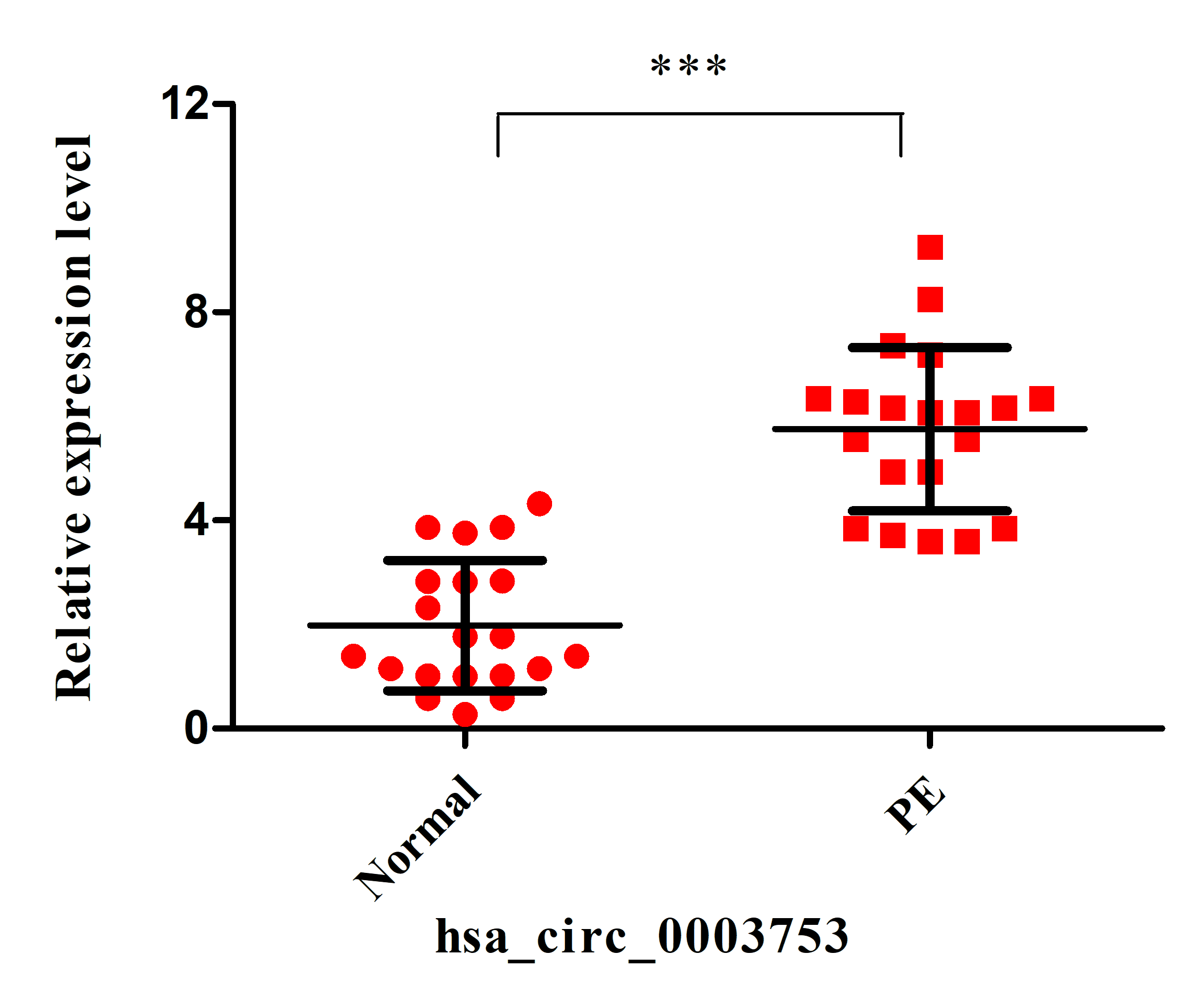

Supplement: Supplemental Information 1 — These data were analyzed for full transcriptome sequencing of patients with preeclampsia and the normal group. [file peerj-09-11299-s001.zip › up-regulated/hsa_circ_0003753.tif]

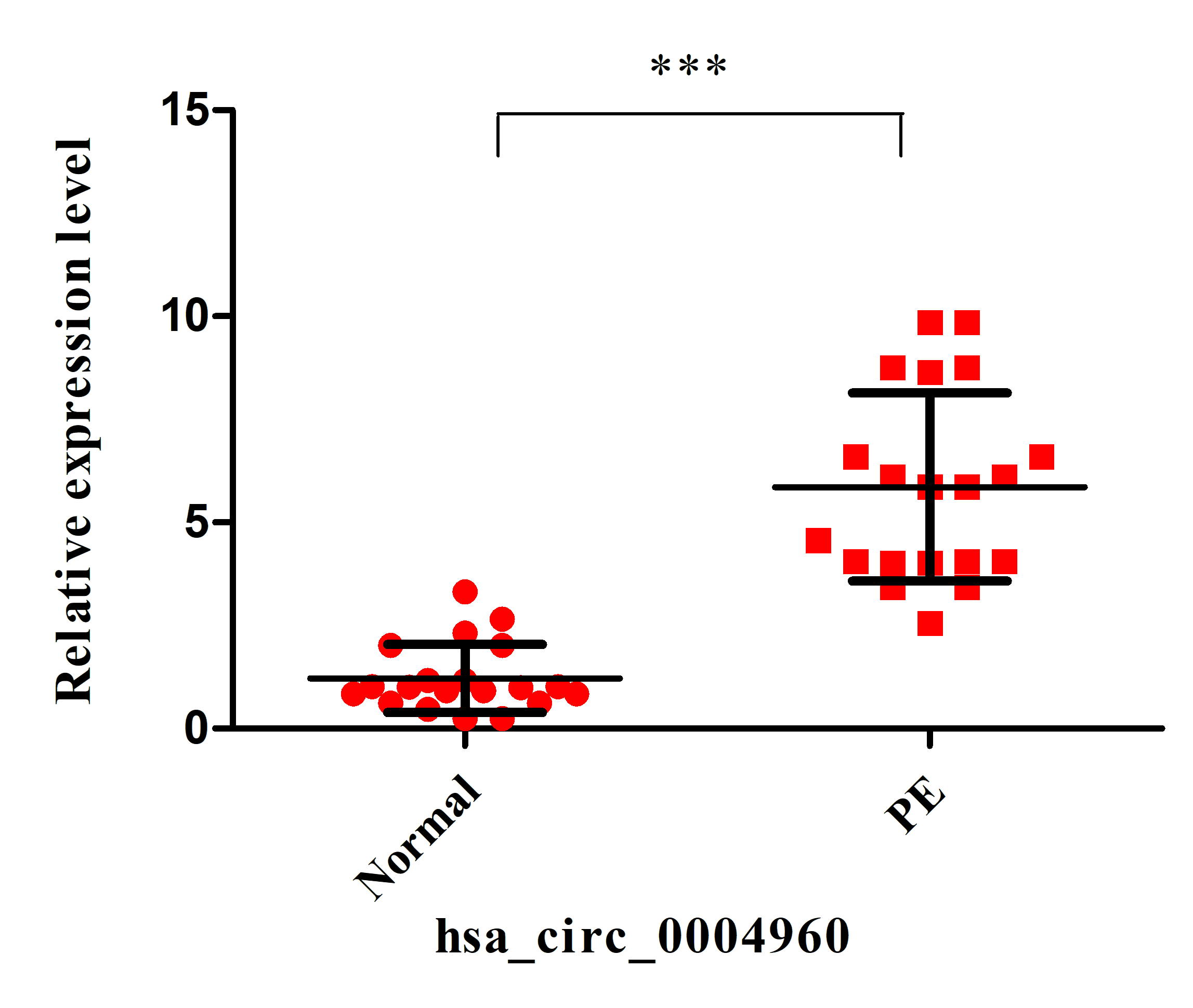

Supplement: Supplemental Information 1 — These data were analyzed for full transcriptome sequencing of patients with preeclampsia and the normal group. [file peerj-09-11299-s001.zip › up-regulated/hsa_circ_0004960.tif]

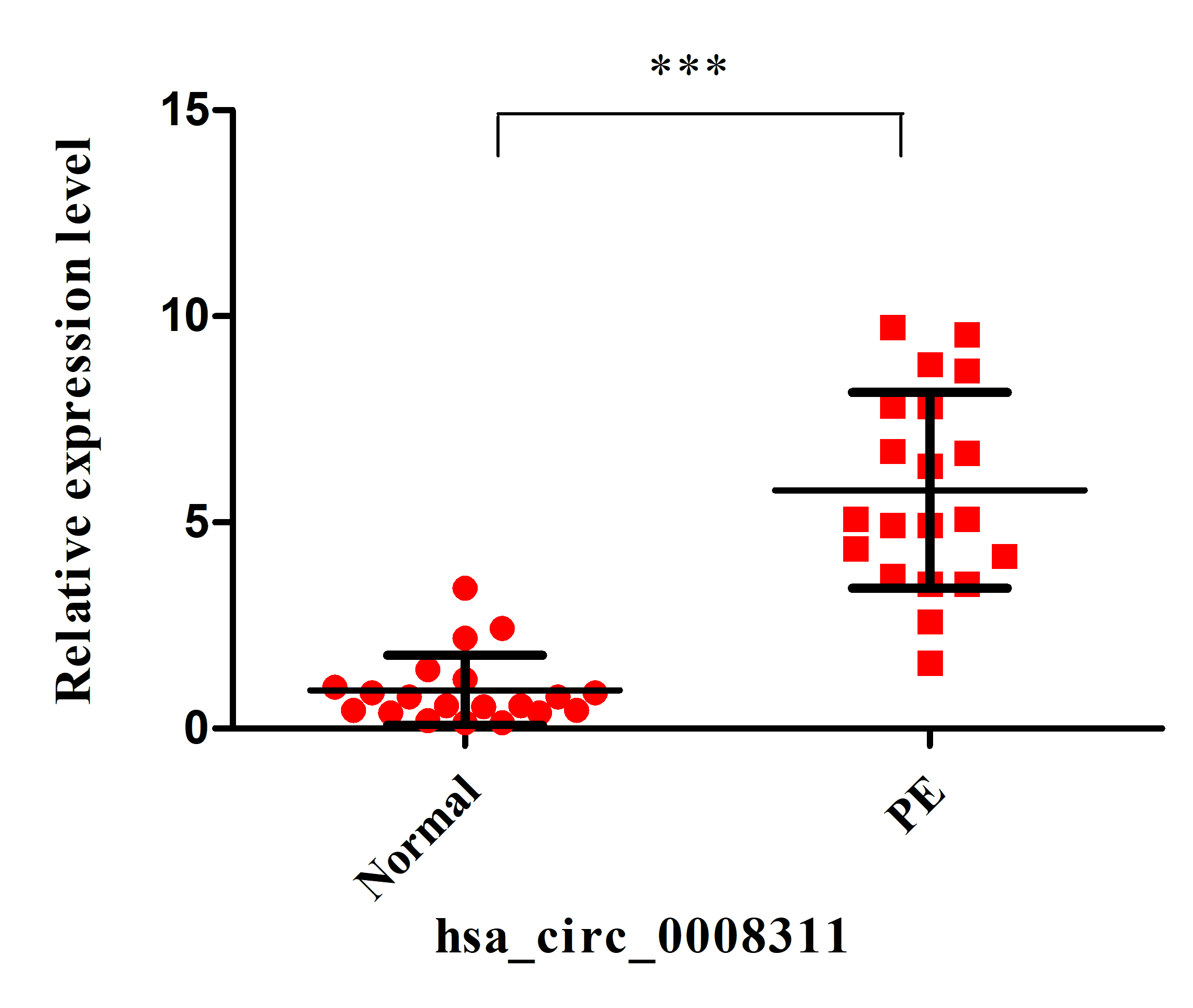

Supplement: Supplemental Information 1 — These data were analyzed for full transcriptome sequencing of patients with preeclampsia and the normal group. [file peerj-09-11299-s001.zip › up-regulated/hsa_circ_0008311.tif]
